# Enzyme-Free Electrochemical Sensors for *in situ* Quantification of Reducing Sugars Based on Carboxylated Graphene–Carboxylated Multiwalled Carbon Nanotubes–Gold Nanoparticle–Modified Electrode

**DOI:** 10.3389/fpls.2022.872190

**Published:** 2022-04-28

**Authors:** Ke Liu, Xiaodong Wang, Bin Luo, Cheng Wang, Peichen Hou, Hongtu Dong, Aixue Li, Chunjiang Zhao

**Affiliations:** ^1^Heyuan Branch, Guangdong Laboratory for Lingnan Modern Agriculture, Heyuan, China; ^2^Research Center of Intelligent Equipment, Beijing Academy of Agriculture and Forestry Sciences, Beijing, China; ^3^College of Landscape and Ecological Engineering, Hebei University of Engineering, Handan, China

**Keywords:** *in situ*, enzyme-free, reducing sugars, carboxylated graphene, carboxylated multi-walled carbon nanotubes, screen-printed electrode

## Abstract

The reducing sugars of plants, including glucose, fructose, arabinose, galactose, xylose, and mannose, are not only the energy source of plants, but also have the messenger function of hormones in signal transduction. Moreover, they also determine the quality and flavor of agricultural products. Therefore, the *in situ* quantification of reducing sugars in plants or agriculture products is very important in precision agriculture. However, the upper detection limit of the currently developed sugar sensor is not high enough for *in situ* detection. In this study, an enzyme-free electrochemical sensor for *in situ* detection of reducing sugars was developed. Three-dimensional composite materials based on carboxylated graphene–carboxylated multi-walled carbon nanotubes attaching with gold nanoparticles (COOH-GR–COOH-MWNT–AuNPs) were formed and applied for the non-enzymatic determination of glucose, fructose, arabinose, mannose, xylose, and galactose. It was demonstrated that the COOH-GR–COOH-MWNT–AuNP-modified electrode exhibited a good catalysis behavior to these reducing sugars due to the synergistic effect of the COOH-GR, COOH-MWNT, and AuNPs. The detection range of the sensor for glucose, fructose, arabinose, mannose, xylose, and galactose is 5–80, 2–20, 2–50, 5–60, 2–40, and 5–40 mM, respectively. To our knowledge, the upper detection limit of our enzyme-free sugar sensor is the highest compared to previous studies, which is more suitable for *in situ* detection of sugars in agricultural products and plants. In addition, this sensor is simple and portable, with good reproducibility and accuracy; it will have broad practical application value in precision agriculture.

## Introduction

Sugars play an important role in plant growth and development. They are not only the energy source of plants, but also have the messenger function of hormones in the process of signal transduction (Li and Sheen, 2016). They also determine the quality and flavor of agricultural products. Moreover, sugar-related materials, such as cellulose, can be developed into eco-friendly and economically favorable biosorbents and biocomposites for removing some toxic substances, such as acid dye (Kamran et al., 2022), CO_2_ (Kamran and Park, 2021a,b), Li^+^ (Kamran and Park, 2020, 2022), and bacteria (Kamran et al., 2019a). Therefore, quantitative analysis of sugar in plants and agricultural products is very important. The traditional methods for determining sugar content include chromatography (Wahjudi et al., 2010), fluorescence method (Pablos et al., 2015), spectrophotometry (Biscay et al., 2012), and colorimetry (Ayyub et al., 2013). However, most of these methods need to be equipped with large-scale instruments and have poor portability (Li et al., 2017). With the development of precision agriculture, researchers often need to conduct *in situ* and on-site detection of the sugar content in plants or agricultural products. Therefore, there is an urgent need to develop new detection methods to achieve in situ and on-site detection of sugars in plants or agricultural products.

Electrochemical biosensor has the advantages of high sensitivity, good selectivity, good portability, fast response, and easy integration. Its development provides an effective solution for in situ and on-site measurement. In recent years, researchers have developed a variety of sugar sensors, such as glucose sensors (Gao et al., 2016; Strakosas et al., 2019) and fructose sensors (Gota et al., 2017; Xu et al., 2018). Compared with enzyme biosensors, enzyme-free biosensors have the advantages of independent of enzyme, less affected by environmental factors, low cost, good stability, and simple preparation (Xu et al., 2014a). For example, Shekarchizadeh et al. (2013) developed an enzyme-free sensor modified with copper oxide nanoparticles and multi-walled carbon nanotubes to improve its electrical activity and selectivity for the detection of glucose and fructose. de Sá et al. (2016) modified glassy carbon electrode with carbon nanotubes and metal hydroxyl oxides to detect and quantitatively analyze carbohydrates (glucose, xylose, galactose, and mannose) in sugarcane. In plants or agricultural products, the content of sugars is very high, ranging from a few millimoles to thousands of millimoles (Zhou et al., 2019). However, most developed glucose sensors are mainly used to detect glucose in humans and animals; their detection range is not suitable for plants. In addition, the highest upper detection limit of the developed sugar sensor is only more than 10 millimoles (Jeong et al., 2018), which is not high enough for *in situ* detection. Therefore, it is necessary to develop new electrochemical sensors for *in situ* detecting sugars in plants or agricultural products.

Carbon-based nanomaterials, such as graphene (GR; Hernaez, 2020), multi-walled carbon nanotubes (MWNT), carbon spherical shells (Campos et al., 2018), and carbon black (Raymundo-Pereira et al., 2017), have received extensive attention in sensor construction due to their extraordinary physical and chemical properties (Kamran et al., 2019b). GR can be obtained by chemical reduction after graphite oxidation, but it is prone to aggregate due to π–π interaction (Shim, 2019). As a special allotrope of GR, MWNT has a unique structure and performance, such as good conductivity, perfect chemical stability, acceleration ability of electron transfer on electrode surface, and large surface area (Özcan et al., 2020a,b). MWCNT- and MWCNT-based nanocomposites have a wide range of applications in the electrochemical field, such as nanosensors (Yola et al., 2021; Yola and Atar, 2021) and fuel cell (Gizem Güneştekin et al., 2020). However, if MWNT cannot be sufficiently dispersed to form a network to meet the electrical conductivity, it will not be able to get a better performance (Zhang et al., 2017). Recent studies have shown that the above problems can be effectively avoided by introducing MWNT between GR nanosheets (Su et al., 2017; Wang et al., 2018). The good dispersion of MWNT can avoid the aggregation of GR flakes. As a surfactant, GR nanoflakes can also directly disperse MWNT to form a three-dimensional (3D) network structure with large specific surface area and excellent electrical conductivity (Cui et al., 2015; Tourani et al., 2015). Moreover, the carboxylated graphene (COOH-GR) and carboxylated multi-walled carbon nanotubes (COOH-MWNT) have better hydrophilicity, biocompatibility, and carboxyl functional groups, which will further improve the sensor performance. Gold (Au) catalysts are attractive nanomaterials due to their excellent photoelectric properties and catalytic activity. Therefore, they are widely used in sensor field, such as glucose oxidation and vitamin detection (Sharma et al., 2020). This catalysis also occurs in other reducing sugars, such as fructose, arabinose, galactose, xylose, and mannose. All these reducing sugars have very similar structures, so the catalytic reaction of Au to them is similar.

Screen-printed electrode (SPE) is widely used because of its low cost, small size, mature manufacturing technology, and good electrochemical performance (Pohanka, 2020). In this study, SPE was used as the basic electrode, and COOH-GR and COOH-MWNT were used to construct a 3D network structure to immobilize AuNPs. Then, this COOH-GR–COOH-MWNT–AuNPs composite material-modified SPE was used to catalyze six reducing sugars (glucose, fructose, arabinose, galactose, xylose, and mannose), and an enzyme-free electrochemical sensor for these six reducing sugars was developed. The upper detection limit of our sensor was improved to 80 millimoles (for glucose), which will have broad application prospect in the *in situ* detection of reducing sugars in plants and agricultural products.

## Materials and Methods

### Chemicals

Carboxyl graphene (GR-COOH) and carboxyl multi-walled carbon nanotube (MWNT-COOH) were purchased from Xianfeng Nanomaterials Technology Co., Ltd. (Nanjing, China). D-glucose, D-fructose, D-galactose, trisodium citrate (C_6_H_5_Na_3_O_7_), citric acid, and anhydrous malic acid were purchased from Sinopharm Chemical Reagent Co., Ltd. Company (Shanghai China). Chloroauric acid (HAuCl_4_), nafion solution (5wt%), D-xylose (xylose), L-arabinose (arabinose), mannose (mannose), D-leucine, DL-tryptophan, lysine, magnesium chloride, sucrose, Betaine, 3-indoleacetic acid, abscisic acid, gibberellins, and ascorbic acid were purchased from Sigma Reagent Co., Ltd. (St. Louis, Missouri, United States). The rest of the reagents are of analytical grade, and ultrapure water was used to prepare the solution throughout the experiment.

### Instruments

Equipped with X-ray energy spectrum analysis (EDS), field emission scanning electron microscopy (FESEM) system (ZEISS SEM 500, Germany), Fourier infrared spectrometer (Thermo Nicolet IS5) were used to study the different modification steps SPE morphology. Glassy carbon sheets were used for SEM, EDS-mapping, and FTIR characterization. The detection of sugars was performed on CHI760E electrochemical workstation (Shanghai Chenhua Instrument Co., Ltd., China). Three-electrode SPEs was used, in which the working electrode and counter electrode are carbon-based and the reference electrode is silver/silver chloride. The diameter of the working electrode is 2.5 mm.

### Preparation of COOH-GR–COOH-MWNT–AuNPs Composite

**Figure 1:**
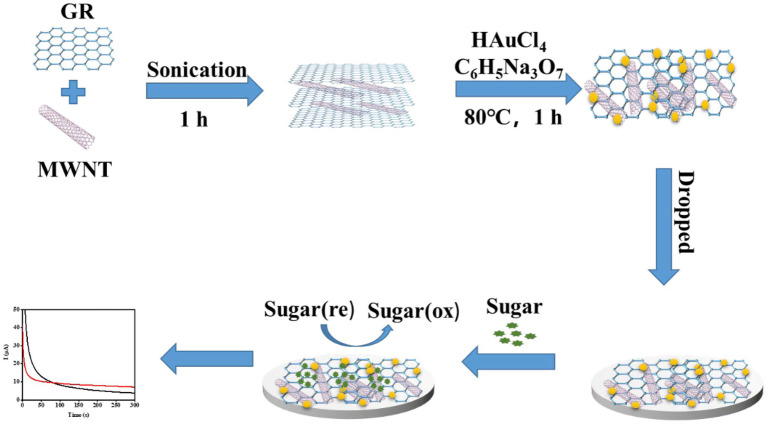
Schematic diagram of preparation process of the enzyme-free sugar sensor.

First, 0.5 mg/ml of COOH-GR and 1.5 mg/ml of COOH-MWNT were mixed. Fifteen milliliters of 23.6 mM HAuCl_4_ solution was added to 20 ml of COOH-GR–COOH-MWNT mixture and stirred magnetically for 60 min. Subsequently, 20 ml of 68 mM trisodium citrate solution was added to the mixture, and the mixture was stirred magnetically for 30 min. Then, this mixture was heated at 80°C for 30 min. The resulting solution was centrifuged at 17,000 rpm for 10 min. Then the precipitate was collected and dried at 60°C for 12 h. 2.5 ml ethanol and 55 μl nafion solution were added to 55 mg dried material. Then, the COOH-GR–COOH-MWNT–AuNPs’ composite was obtained. Four microliters of the COOH-GR–COOH-MWNT–AuNPs solution was used to modify the SPE electrode by dropping method. The modification process is shown in Figure 1.

### Measurement Procedure

Cyclic voltammetry (CV) was used to study the catalytic effect of the modified electrode toward different sugars, the scanning range was −0.6 to 0.6 V, and the scanning speed was 0.05 V/s. The concentration of sugars was detected by chronoamperometry (i-t); the working voltage is 0.3 V. All electrochemical tests were carried out in 0.1 M NaOH solution.

### Sugars Determination in Apple Juice by Traditional Analytical Methods

Reference control measurement of glucose and fructose concentration in apple juice was carried out by high-pressure liquid chromatography (HPLC). For arabinose, mannose, xylose, and galactose, they cannot be separated by the HPLC method. So the ion chromatography (IC) method was used to measure these sugars. The apple juice was bought from local supermarket. The apple juice was filtered through a nylon filter (aperture 0.45 μm). Glucose and fructose were determined by Agilent chromatograph. Arabinose, mannose, xylose, and galactose were determined by the ICS-3000 chromatograph. They were separated on an amino column and eluted with 78% acetonitrile solution. Glucose and fructose were detected by PA1 differential refractive index detector, and the other four sugars were detected by amperometric pulse detector.

## Results and Discussion

### Morphology and Structure Characterization of the Sensor

**Figure 2:**
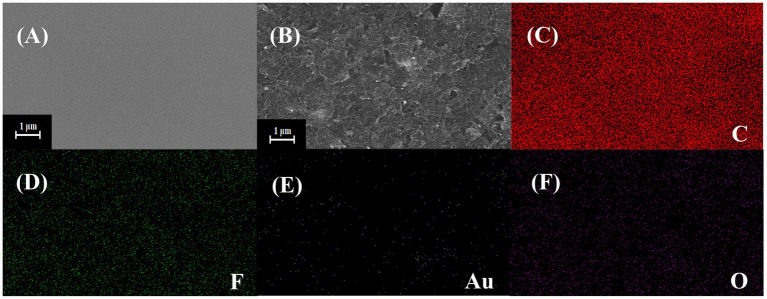
SEM images of **(A)** bare SPE, **(B)** COOH-GR–COOH-MWNT–AuNPs/SPE, **(C–F)** are the EDS mapping images of COOH-GR–COOH-MWNT–AuNPs/SPE.

Figure 2 shows the result of SEM. It can be seen from Figure 2A that the surface of the bare SPE is smooth without any impurities. Figure 2B shows the modified SPE. The lamellar wrinkled COOH-GR structure can be observed, tubular COOH-MWNT intersperse between COOH-GR nanosheets, and gold nanoparticles are distributed in the COOH-GR–COOH-MWNT. The size of gold nanoparticles is about 30–50 nm. Figures 2C–F shows the EDS mapping spectrum after the SPE was modified with COOH-GR–COOH-MWNT–AuNPs, and the signals of C, F, Au, and O elements are obtained. The existence of C element is attributed to the C element in COOH-GR, COOH-MWNT, and glassy carbon sheet. Since nafion contains a large amount of F element, it leads to the emergence of F element. The O elements are derived from graphene, and the Au in COOH-GR–COOH-MWNT–AuNPs is the reason for the appearance of Au element. The results of SEM and EDS proved that COOH-GR–COOH-MWNT–AuNP materials have been successfully modified on the electrode surface.

Supplementary Figure S1 shows the bare and COOH-GR–COOH-MWNT–AuNP images by FTIR. Both C=O at 1,720 cm^−1^ and O-H at 3,346 cm^−1^ are characteristic peaks of carboxyl groups, which attribute to the COOH-GR and COOH-MWNT. Glassy carbon sheet was used as the substrate for this experiment. It is well known that glassy carbon sheet comprises thin, tangled ribbons of cross-linked graphite-like sheets that share sp2 bonding and the basic structure of a six-member ring (Shi and Shiu, 2002). But the cross-linked six-member rings are possibly broken up at the surface in the polishing process. Moieties containing alcohol, phenol, aldehyde, ketone (or quinine), and carboxylic acid (or anhydride) are appeared to connect to the skeleton of glassy carbon sheet (Wu et al., 2008). Therefore, there was no significant difference between the bare and COOH-GR–COOH-MWNT–AuNP images. The effect of Au nanoparticles in FTIR spectrum cannot be observed, since they do not have molecular bonds. Similar results were also observed by Tabatabaie and Dorranian (2016) and Najafianpour and Dorranian (2018).

### Electrochemical Characterization of the Sensor Preparation Process

**Figure 3:**
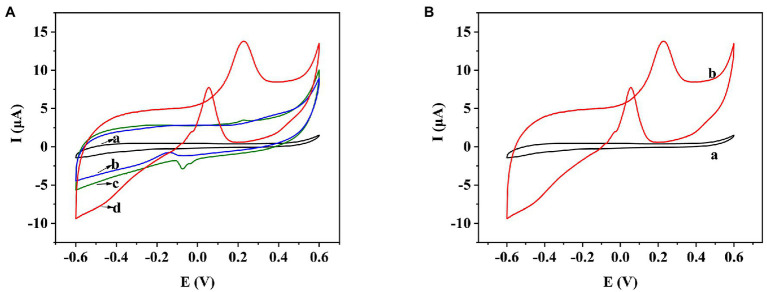
**(A)** CV behavior of bare SPE (a), COOH-GR/SPE (b), COOH-MWNT/SPE (c), and COOH-GR–COOH-MWNT–AuNPs/SPE (d) in 20 mM glucose. **(B)** CV behavior of COOH-GR–COOH-MWNT–AuNPs/SPE with and without 20 mM glucose.

First, the preparation process of the sensor was characterized by the CV method (Supplementary Figure S2A). The CV scan was performed in a 5 mM [Fe(CN)_6_]^3−/4−^ solution (containing 0.1 M KCL). When the COOH-GR–COOH-MWNT–AuNPs material was modified on the electrode, the redox peak current increased and the peak-to-peak potential difference decreased. This is due to that the high conductivity and catalytic performance of COOH-GR, COOH-MWNT, and AuNPs increase the electron transfer rate and enhance the reversibility of the electrode. Consistent with that of CV, the Nyquist curve (Supplementary Figure S2B) of the bare electrode has a smaller half arc, while there is almost no half arc after the electrode was modified with COOH-GR–COOH-MWNT–AuNPs nanocomposite. After fitting with a simple equivalent circuit model (inset in Figure 3B), the interfacial electron transfer resistance Rct can be obtained. The Rct value of GR-MWNT-Au/SPE (1,038 Ω) is lower than that of bare electrode (508.4 Ω), which also attributes to the high conductivity and catalytic performance of GR and MWNT. The results of CV and EIS both prove that the preparation of the sensor is successful and effective. Supplementary Figure S2C shows the CV graph of COOH-GR–COOH-MWNT–AuNPs/SPE in 5 mM [Fe(CN)_6_]^3−/4−^ solution (containing 0.1 M KCL) at various scan rates. The effective surface area of the different modified SPE was evaluated based on the Randles–Sevcik equation (Xu et al., 2014b):


$$I_{P}=2.69\times 10^{5}\times n^{3/2}AD_{0}^{1/2}Cv^{1/2}$$

where *D*_0_ is the diffusion coefficient of the molecule in solution (cm^2^ s^−1^), A is the effective area of the electrode (cm^2^), *v* is the scan rate (V s^−1^), *n* is the number of electrons including in the redox reaction, and C_0_ is the concentration of the probe in the solution (mol cm^−3^). For [Fe(CN)_6_]^3−^/[Fe(CN)_6_]^4−^, *n* = 1, C_0_ = 5 × 10^−6^ mol cm^−3^, *D*_0_ = 1 × 10^−5^ cm^2^ s^−1^ (Wang et al., 2012). The effective surface area was 0.1041 cm^2^ for the COOH-GR–COOH-MWNT–AuNPs/SPE, respectively, which was much higher than that of bare SPE (0.030 cm^2^).

### Electrochemical Performance of COOH-GR–COOH-MWNT–AuNP Nanomaterials

In order to test the electrochemical performance of COOH-GR–COOH-MWNT–AuNP nanomaterials, the electrochemical behaviors of bare SPE, COOH-GR–AuNPs/SPE, COOH-MWNT–AuNPs/SPE and COOH-GR–COOH-MWNT–AuNPs/SPE in the range of −0.6 to 0.6 V were investigated. Taking glucose as an example, the CV scan in Figure 3A was performed in 20 mM glucose (containing 0.1 M NaOH). The bare SPE electrode (curve a) does not show any oxidation peak in the range of −0.6 to 0.6 V. Because COOH-GR, COOH-MWNT, and AuNPs are all highly conductive, which can improved the electrochemical catalytic behavior of the sensor, an oxidation peak was appeared at about 0.3 V in GR-Au/SPE (curve b) and MWNT-Au/SPE (curve c), which was due to the oxidation of glucose. The highest oxidation peak was obtained on COOH-GR–COOH-MWNT–AuNPs/SPE at about 0.3 V (curve d), which indicates the synergistic effect of the COOH-GR, COOH-MWNT, and AuNPs in the catalysis of glucose. Therefore, COOH-GR–COOH-MWNT–AuNPs material was chosen for subsequent experiments. Figure 3B shows that there is no oxidation peak in COOH-GR–COOH-MWNT–AuNPs/SPE without the addition of glucose (curve a), while an obvious oxidation peak was observed at about 0.3 V after adding 20 mM glucose (b). This result also confirms that the appearance of oxidation peak is due to the oxidation of glucose, not the COOH-GR, COOH-MWNT, or Au nanoparticles. This catalysis also occurs in other sugars. In the presence of fructose, galactose, arabinose, mannose, or xylose, respectively, similar signals to glucose can be observed. Since these sugars have very similar structures, they are monosaccharides containing aldehyde or ketone groups, the COOH-GR–COOH-MWNT–AuNP nanomaterials have similar catalytic effect to these sugars (Xu et al., 2014a). The modified electrode can catalyze the oxidation of these sugars to form corresponding esters, which are hydrolyzed to form acids (Wang et al., 2020).

### Optimization of Sensor Preparation Conditions

**Figure 4:**
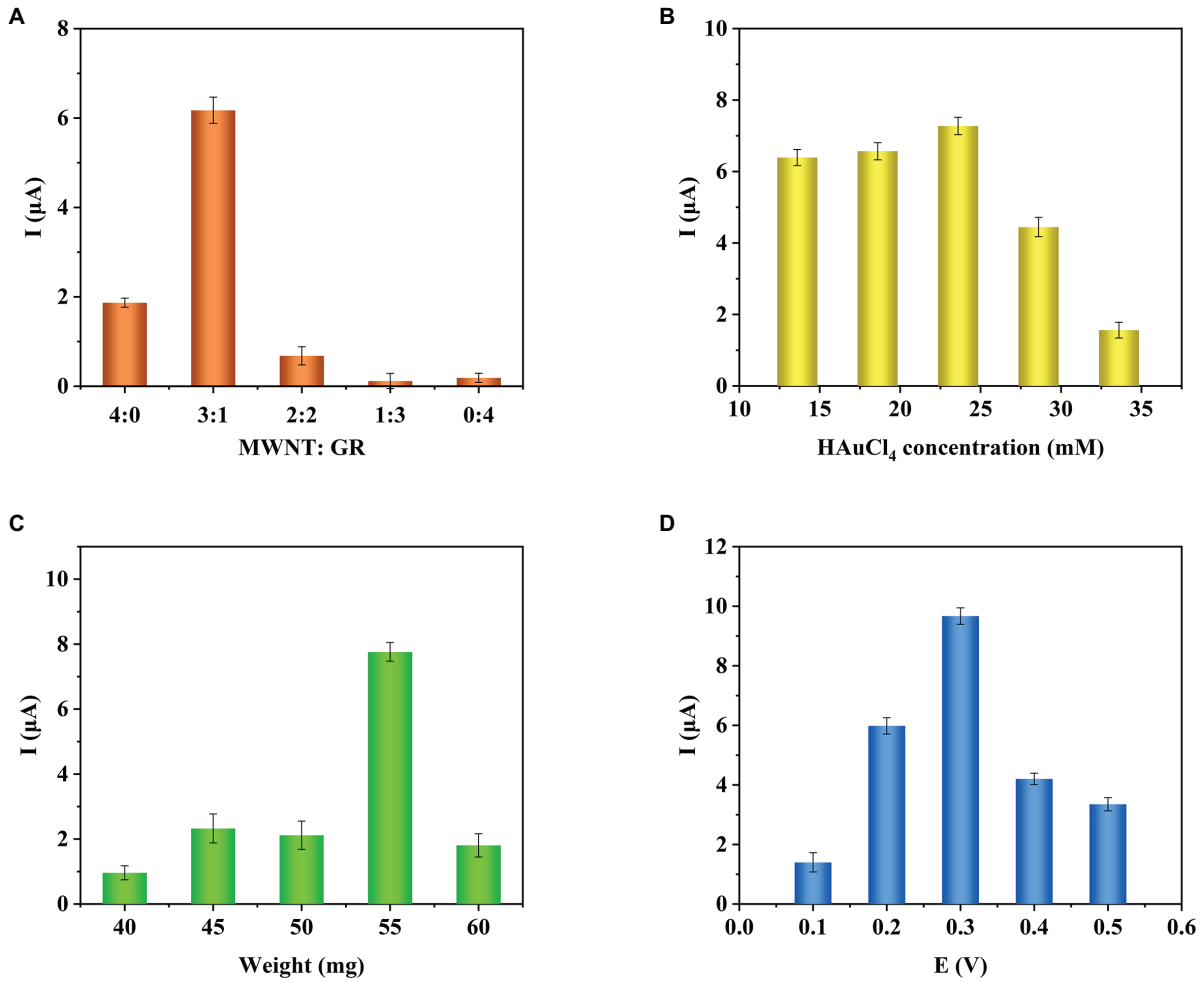
Effect of COOH-GR–COOH-MWNT content ratio **(A)**, HAuCl_4_ concentration **(B)**, total weight of dropped composite material **(C)**, working voltage **(D)** on the current response.

In order to achieve the best performance of the sensor, the conditions for preparing the sensor were optimized. In this study, the total amount of immobilized carbon nanomaterials was 2 mg/ml. The effect of single COOH-GR, COOH-MWNT, and COOH-GR-COOH-MWNT composite materials with different ratios (3:1, 2:2, 1:3) on the response current was investigated. The i-t response was measured using a glucose concentration of 20 mM. As shown in Figure 4A, in the various ratios of COOH-GR–COOH-MWNT composites, the maximum current response is obtained when COOH-GR: COOH-MWNT is 1:3, which shows that COOH-GR and COOH-MWNT have the best synergistic effect at this ratio. Therefore, the optimal content of COOH-GR and COOH-MWNT in this study is 0.5 and 1.5 mg/ml, respectively.

Gold nanoparticles are one of the important components of the composite material, so it is necessary to optimize the concentration of HAuCl_4_ (13.6, 18.6, 23.6, 28.6, 33.6 mM). The optimization results are shown in Figure 4B. It can be seen that when the HAuCl_4_ content is from 13.6 to 23.6 mM, the current response gradually increases. When the HAuCl_4_ content continues to increase, the response current no longer increases, so 23.6 mM was selected for HAuCl_4_ concentration for the subsequent experiments.

The total weight of the dropped composite material affects the performance of the modified electrode, which also requires optimization (40, 45, 50, 55, and 60 mg). The results are shown in Figure 4C. When the total weight of the material is 55 mg, the response current is highest. Therefore, the total weight of the composite material is determined to be 55 mg.

Finally, the influence of different voltages (0.1, 0.2, 0.3, 0.4, and 0.5 V) on the response current was examined. As shown in Figure 4D, when the voltage increases from 0.1 to 0.3 V, the response current gradually increases. When the voltage continued to increase, the current dropped. So 0.3 V is the best voltage for the catalytic reaction of glucose.

### Performance of the Enzyme-Free Sugar Sensor

**Figure 5:**
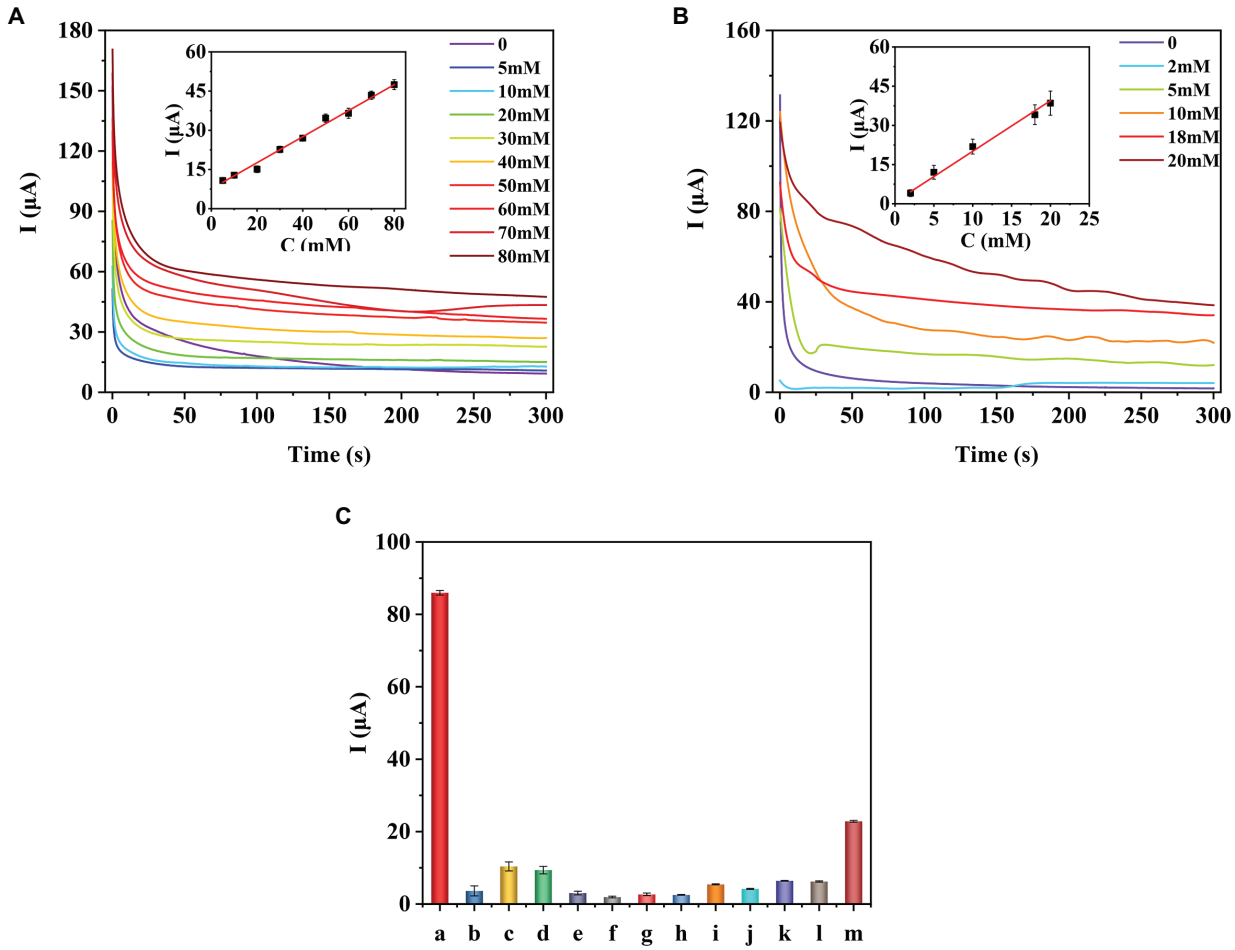
I–t curves and calibration curves of the COOH-GR–COOH-MWNT–AuNPs/SPE sensor for the detection of different concentrations of glucose **(A)** and fructose **(B)**. Selectivity of the prepared the sugar sensor **(C)**. a-glucose, b-malic acid, c-citric acid, d-tryptophan, e-leucine, f-lysine, g-magnesium chloride, h-sucrose, i-Betaine, j-3-indoleacetic acid, k-abscisic acid, l-gibberellins, m-ascorbic acid.

Under the optimal experimental conditions, the prepared enzyme-free sugars sensor was used to measure a series of concentrations of glucose, fructose, arabinose, mannose, xylose, or galactose solutions, respectively. The i-t curve for the detection of different concentrations of glucose is shown in Figure 5A. The results of linear fitting are shown in the inset of Figure 5A. The sensor has a linear relationship between glucose concentration and response current in the range of 5–80 mM. The linear equation is *I*(μA) = 7.268 + 0.507C (mM), the correlation coefficient *R*^2^ = 0.9911, and the detection limit (LOD) is 0.537 μM (S/N = 3). As for fructose (Figure 5B), the detection range of the sensor is 2–20 mM, the linear equation is *I*(μA) = 0.720 + 1.936C (mM), and the LOD is 1.630 μM. The sensor also showed similar current response to arabinose, galactose, mannose, and xylose (Supplementary Figures S3–S6). Table 1 shows the analysis characteristics of the sensor to all sugars. In plants or agricultural products, the content of sugars is very high, ranging from a few millimoles to thousands of millimoles (Zhou et al., 2019). Therefore, for *in situ* detection of sugars in plants or agricultural products, the lower detection limit does not need to be as low as micromolar level, while the upper detection limit needs to be as high as tens or even thousands of millimol level. Table 2 shows the analytical performance of different enzyme-free glucose sensors reported previously. From Table 2, we can see that the highest upper detection limit of the developed enzyme-free glucose sensor is 19.6 mM (Jeong et al., 2018). The detection range for glucose of our sensor is 5–80 mM. Clearly, our sensor can be used for in situ detection of glucose in more plants and agricultural products. Supplementary Table S1 shows the comparison of analytical performance of different enzyme-free fructose sensors. Our sensor shows the highest upper detection limit for fructose. Supplementary Table S2 shows the analytical performance of enzyme-free sensors for arabinose, mannose, xylose, and galactose. The upper detection limit of our sensor (Table 1) for these four sugars is all higher than the corresponding reported sensors. Therefore, our sensor is also more suitable for in situ detection of fructose, arabinose, mannose, xylose, and galactose in agricultural products and plants.

**Table 1:** Analytical characteristics of different sugars detected by the sugar sensor.

Sugar	Lineal range (mM)	Intercept	Slope	Calibration	*R* ^2^	LOD (mM)
Glucose	5–80	7.268 ± 0.635	0.507 ± 0.018	7.268 + 0.507C	0.9911	0.537
Fructose	2–20	0.720 ± 0.872	1.936 ± 0.112	0.720 + 1.936C	0.9901	1.630
Arabinose	2–50	3.905 ± 2.792	2.019 ± 0.086	3.905 + 2.019C	0.9910	1.811
Mannose	5–60	−13.812 ± 2.020	30.924 ± 1.542	−13.812 + 30.924lgC	0.9853	4.903
Xylose	2–40	1.270 ± 0.385	8.979 ± 0.390	1.270 + 8.979C	0.9888	0.693
Galactose	5–40	−16.943 ± 4.239	74.263 ± 3.360	−16.943 + 74.263lgC	0.9919	2.105

**Table 2:** Comparison of analytical performance of different enzyme-free glucose sensors.

Electrode	Linear range (mM)	Detection limit (μM)	References
CNTs/AuNPs/GCE	0.002–19.6	0.5	Jeong et al., 2018
Cu/Ni/graphene/Ta	5 × 10^−6^–2.174	0.0027	Cui et al., 2019
CuO/Nafion/GC	0.001–10	0.57	Pérez-Fernández et al., 2017
SPE/NiCo/C	5 × 10^−4^–4.38	0.2	Wang et al., 2020
CuO/Ni(OH)_2_/CC	0.05–8.50	0.31	Sun et al., 2020
MOF/CuO	0–6.535	0.15	Luo et al., 2020
Cu/Ni/Au	5 × 10^−4^–3.0, 3.0–7.0	0.1	Liu et al., 2020
CuO NWs/GC	0.0125–4.29	4.17	Zhang, 2019
Au/CQDs	0.05–3	20	Han et al., 2020
COOH-GR-COOH-MWNT-AuNPs/SPE	5–80	540	This work

In order to test the selectivity of the sensor, the prepared sensor was used to detect different interferences (20 mM), including malic acid, citric acid, tryptophan, leucine, lysine, magnesium chloride, sucrose, betaine, 3-indoleacetic acid, abscisic Acid, gibberellins, and ascorbic acid. The results are shown in Figure 5C, and the current response of the sensor to glucose is significantly higher than other interfering substances, which proves that the sensor has good selectivity.

Under the same experimental conditions, the same SPE electrode was used to continuously measure the glucose solution of the same concentration (20 mM) for five times, and it can be seen (Supplementary Figure S7A) that the current response is relatively consistent (RSD = 7.36%). Five electrodes were used to measure the same concentration of glucose (Supplementary Figure S7B), and the RSD of the response current was 7.49%. These results show that the sensor has excellent reproducibility and high stability.

### Detection in Real Samples

The standard addition method was adopted to detect glucose in apple juice. After 20 times of dilution, different concentrations of glucose were added. The results are shown in Table 3. The recovery results of other sugar are shown in Supplementary Tables S3–S7. The spiked recovery rate of glucose, fructose, arabinose, mannose, xylose, and galactose is 97.40–100.85%, 101.28–105.42%, 96.69–105.28%, 97.73–105.12%, 99.91–104.78%, and 98.42–104.89%, respectively, which show that our sensor has good practicality. Moreover, the results were also compared with those obtained by other methods. The initial concentration of glucose and fructose in the apple juice was also detected by the HPLC methods. The relative error of the results obtained by the as-prepared sensor and HPLC methods was 11.81 and 14.70%. The relative error between these two methods was smaller than 15%, which is considered acceptable (Artigues et al., 2021). For arabinose, mannose, xylose, and galactose, they cannot be separated by HPLC method. So the IC method was used to measure these sugars. As their content is very low, which has exceeded the detection range of IC, so the comparison result cannot be obtained. But the initial concentration of arabinose and mannose can be detected by our sensor, indicating that the as-prepared sensor has more potential in practical applications.

**Table 3:** Recovery rate of glucose in apple juice (*n* = 3).

Glucose initial (mM)	Added (mM)	Found (mM)	RSD (%)	Recovery (%)
12.075 (sensor)	10	22.092	3.45	100.17
10.800 (HPLC)	20	32.244	6.99	100.85
	30	41.294	5.69	97.40

## Conclusion

In summary, the developed enzyme-free reducing sugar sensor catalyzes the oxidation reaction of six sugars through the synergistic effect of graphene, carbon nanotubes, and gold nanoparticles. The detection range of the sensor for glucose, fructose, arabinose, mannose, xylose, and galactose is 5–80, 2–20, 2–50, 5–60, 2–40, and 5–40 mM, respectively. To our knowledge, the upper detection limit of our enzyme-free sugar sensor is the highest compared to previous studies, which is more suitable for in-situ detection of sugars in agricultural products and plants. This sensor is simple and portable and has good reproducibility and stability. Therefore, it will have broad practical application value in precision agriculture. With the introduction of various new nanomaterials, sensors with wider detection range, higher upper detection limit and better selection performance are expected to be developed, which is more suitable for the *in situ* quantification of sugars in plants and agricultural products.

## Data Availability Statement

The original contributions presented in the study are included in the article/**Supplementary Material**, further inquiries can be directed to the corresponding authors.

## Author Contributions

KL and XW performed the experiments, analyzed the data, and wrote the original manuscript. BL, CW, PH, and HD helped to perform the experiments. AL and CZ supervised the project, designed the research, and wrote, reviewed, and edited the manuscript. All authors contributed to the article and approved the submitted version.

## Funding

The authors are thankful for the funding from the Key-Area Research and Development Program of Guang Dong Province (No. 2021B0707010002) and the National Natural Science Foundation of China (Grant No. 21974012).

## Conflict of Interest

The authors declare that the research was conducted in the absence of any commercial or financial relationships that could be construed as a potential conflict of interest.

## Publisher’s Note

All claims expressed in this article are solely those of the authors and do not necessarily represent those of their affiliated organizations, or those of the publisher, the editors and the reviewers. Any product that may be evaluated in this article, or claim that may be made by its manufacturer, is not guaranteed or endorsed by the publisher.
