# Supplementary material for: Enzyme-Free Electrochemical Sensors for in situ Quantification of Reducing Sugars Based on Carboxylated Graphene–Carboxylated Multiwalled Carbon Nanotubes–Gold Nanoparticle–Modified Electrode
Source: Front Plant Sci. 2022 Apr 28;13:872190. doi: 10.3389/fpls.2022.872190 (PMC9098227; doi:10.3389/fpls.2022.872190)
Supplement: Supplementary file 1 [file Data_Sheet_1.docx]

Supplementary Material

# Supplementary Figures

**Figure S1.** CV graph (A) and EIS graph (B) of bare SPE (a) and GR-MWNT-Au/SPE (b) in 5 mM [Fe(CN)6]3−/4− solution (containing 0.1 M KCL).

**Figure S2.** i-t curves of GR-MWNT-Au/SPE sensor for detection of different concentrations of arabinose, (B) The calibration curves of arabinose in the range of 2 mM to 50 mM.

**Figure S3.** i-t curves of GR-MWNT-Au/SPE sensor for detection of different concentrations of mannose, (B) The calibration curves of mannose in the range of 5 mM to 60 mM. ****

**Figure S4.** i-t curves of GR-MWNT-Au/SPE sensor for detection of different concentrations of xylose, (B) The calibration curves of xylose in the range of 2 mM to 40 mM.

**Figure S5.** i-t curves of GR-MWNT-Au/SPE sensor for detection of different concentrations of galactose, (B) The calibration curves of galactose in the range of 5 mM to 40 mM.

**Figure S6.** (A) i-t curves of the same electrode for 5 times consecutive experiments in 20 mM glucose, (B) i-t curves of 5 electrodes in 20 mM glucose.
