# Supplementary material for: Enzyme-Free Electrochemical Sensors for in situ Quantification of Reducing Sugars Based on Carboxylated Graphene–Carboxylated Multiwalled Carbon Nanotubes–Gold Nanoparticle–Modified Electrode
Source: Front Plant Sci. 2022 Apr 28;13:872190. doi: 10.3389/fpls.2022.872190 (PMC9098227; doi:10.3389/fpls.2022.872190)
Supplement: Supplementary file 2 [file Data_Sheet_2.docx]

Supplementary Material

# Supplementary Figures

**Figure S1.** FTIR curves of bare and COOH-GR-COOH-MWNT-AuNPs.

**Figure S2.** CV graph (A) and EIS graph (B) of bare SPE (a) and COOH-GR-COOH-MWNT-AuNPs/SPE (b). CV graph (C) of different scan rates of COOH-GR-COOH-MWNT-AuNPs/SPE in 5 mM [Fe(CN)_6_]^3−/4−^ solution (containing 0.1 M KCL).

**Figure S3.** i-t curves of COOH-GR-COOH-MWNT-AuNPs/SPE sensor for detection of different concentrations of arabinose, (B) The calibration curves of arabinose in the range of 2 mM to 50 mM.

**Figure S4.** i-t curves of COOH-GR-COOH-MWNT-AuNPs/SPE sensor for detection of different concentrations of mannose, (B) The calibration curves of mannose in the range of 5 mM to 60 mM. ****

**Figure S5.** i-t curves of COOH-GR-COOH-MWNT-AuNPs/SPE sensor for detection of different concentrations of xylose, (B) The calibration curves of xylose in the range of 2 mM to 40 mM.

**Figure S6.** i-t curves of COOH-GR-COOH-MWNT-AuNPs/SPE sensor for detection of different concentrations of galactose, (B) The calibration curves of galactose in the range of 5 mM to 40 mM.

**Figure S7.** (A) i-t curves of the same electrode for 5 times consecutive experiments in 20 mM glucose, (B) i-t curves of 5 electrodes in 20 mM glucose.

# Supplementary Tables

**Table S1 Comparison of analytical performance of different enzyme-free fructose sensors.**

| Electrode | Linear range  (mM) | Detection limit  (μM) | Ref. |
| --- | --- | --- | --- |
| ZnO/CdO/SnO2/GCE | 1×10^-7^-0.01 | 9.589×10^7^ | (Alam et al., 2020) |
| Co_3_O_4_/FTO | 0.021-15 | 1.7 | (Gota et al., 2017) |
| 4MPB/PtNPs/MWCNT/Au | 2.5-10 | - | (Silva-Carrillo et al., 2017) |
| CuNP/Gr/silica capillary | 0.001-0.02 | 0.51 | (Chen et al., 2012) |
| Co/Au | 0.001-0.01 | 0.005 | (Lang et al., 2013) |
| CuO/Co_3_O_4_/GCE | 0.01-3 | 3 | (Wang et al., 2011) |
| LaMnO_3_/GCE | 0.4-4 | 63 | (Xu et al., 2014) |
| COOH-GR-COOH-MWNT-AuNPs/SPE | 2-20 | 1630 | This work |

**Table S2 Comparison of analytical performance of different enzyme-free sugar sensors.**

| Sugar | Electrode | Linear range  (mM) | Detection limit  (μM) | Ref. |
| --- | --- | --- | --- | --- |
| Arabinose | Cu nanospheres/SPCE | 1-10 | 0.57 | (Pérez-Fernández et al., 2017) |
| Mannose | FcBA/3APBA/4MBA/AuNPs/ITO | 0.5-30 | 43 | (Chen et al., 2021) |
| Xylose | GCE/RGO-MIP | 1×10^-11^-1×10^-8^ | 8×10^-11^ | (Pompeu Prado Moreira et al., 2020) |
| Galactose | NPG | 0.01-1.8 | 5 | (Mie et al., 2020) |

**Table S3 Recovery rate of fructose in apple juice (n = 3).**

| Fructose initial  （mM） | Added  （mM） | Found  （mM） | RSD  （%） | Recovery  （%） |
| --- | --- | --- | --- | --- |
| 17.491 (sensor) | 5 | 22.555 | 5.36% | 101.28% |
| 15.250 (HPLC) | 10 | 27.708 | 5.91% | 102.17% |
|  | 20 | 38.575 | 4.39% | 105.42% |

**Table S4 Recovery rate of Arabinose in apple juice (n = 3).**

| Arabinose initial | Added  （mM） | Found  （mM） | RSD  （%） | Recovery  （%） |
| --- | --- | --- | --- | --- |
| 4.698 mM(sensor) | 10 | 15.226 | 4.349% | 105.28% |
|  | 20 | 25.597 | 3.951% | 104.50% |
|  | 30 | 33.704 | 6.868% | 96.69% |

**Table S5 Recovery rate of Mannose in apple juice (n = 3).**

| Mannose initial | Added  （mM） | Found  （mM） | RSD  （%） | Recovery  （%） |
| --- | --- | --- | --- | --- |
| 7.568 mM(sensor) | 10 | 17.455 | 5.618% | 98.87% |
|  | 20 | 27.113 | 4.357% | 97.73% |
|  | 30 | 39.103 | 4.672% | 105.12% |

**Table S6 Recovery rate of Xylose in apple juice (n = 3).**

| Added  （mM） | Found  （mM） | RSD  （%） | Recovery  （%） |
| --- | --- | --- | --- |
| 10 | 10.095 | 5.296% | 100.95% |
| 20 | 19.983 | 4.381% | 99.91% |
| 30 | 31.435 | 4.747% | 104.78% |

**Table S7 Recovery rate of Galactose in apple juice (n = 3).**

| Added  （mM） | Found  （mM） | RSD  （%） | Recovery  （%） |
| --- | --- | --- | --- |
| 10 | 11.842 | 3.418% | 98.42% |
| 20 | 22.977 | 3.753% | 104.89% |
| 30 | 31.979 | 4.992% | 103.26% |

# References

Alam, M.M., Asiri, A.M., Rahman, M.M., and Islam, M.A. (2020). Fabrication of sensitive D-fructose sensor based on facile ternary mixed ZnO/CdO/SnO2 nanocomposites by electrochemical approach. *Surfaces and Interfaces* 19, 100540. doi: 10.1016/j.surfin.2020.100540

Chen, M., Cao, X., Chang, K., Xiang, H., and Wang, R. (2021). A novel electrochemical non-enzymatic glucose sensor based on Au nanoparticle-modified indium tin oxide electrode and boronate affinity. *Electrochimica Acta* 368, 137603. doi: 10.1016/j.electacta.2020.137603

Chen, Q., Zhang, L., and Chen, G. (2012). Facile preparation of Graphene-Copper nanoparticle composite by in situ chemical reduction for electrochemical sensing of carbohydrates. *Analytical Chemistry* 84, 171-178. doi: 10.1021/ac2022772

Gota, T., Chowdhury, M., and Ojumu, T. (2017). Non-enzymatic Fructose Sensor Based on Co3 O4 Thin Film. *Electroanalysis* 29, 2855-2862. doi: 10.1002/elan.201700503

Lang, X., Fu, H., Hou, C., Han, G., and Yang, P., et al. (2013). Nanoporous gold supported cobalt oxide microelectrodes as high-performance electrochemical biosensors. *Nature Communications* 4. doi: 10.1038/ncomms3169

Mie, Y., Katagai, S., and Ikegami, M. (2020). Electrochemical oxidation of monosaccharides at nanoporous gold with controlled atomic surface orientation and Non-Enzymatic galactose sensing. *Sensors* 20, 5632. doi: 10.3390/s20195632

Pérez-Fernández, B., Martín-Yerga, D., and Costa-García, A. (2017). Galvanostatic electrodeposition of copper nanoparticles on screen-printed carbon electrodes and their application for reducing sugars determination. *Talanta* 175, 108-113. doi: 10.1016/j.talanta.2017.07.026

Pompeu Prado Moreira, L.F., Buffon, E., and Stradiotto, N.R. (2020). Electrochemical sensor based on reduced graphene oxide and molecularly imprinted poly(phenol) for d-xylose determination. *Talanta* 208, 120379. doi: 10.1016/j.talanta.2019.120379

Silva-Carrillo, C., Reynoso-Soto, E.A., Paraguay-Delgado, F., Alonso-Núñez, G., and Félix-Navarro, R.M. (2017). Synthesis of PtNPs/MWCNT functionalized with 4-Mercaptophenylboronic acid for an electrochemical sensor of fructose. *Journal of the Electrochemical Society* 164, B86-B91. doi: 10.1149/2.0211704jes

Wang, Y., Wang, W., and Song, W. (2011). Binary CuO/Co3O4 nanofibers for ultrafast and amplified electrochemical sensing of fructose. *Electrochimica Acta* 56, 10191-10196. doi: 10.1016/j.electacta.2011.09.001

Xu, D., Luo, L., Ding, Y., Jiang, L., and Zhang, Y., et al. (2014). A novel nonenzymatic fructose sensor based on electrospun LaMnO3 fibers. *Journal of Electroanalytical Chemistry* 727, 21-26. doi: 10.1016/j.jelechem.2014.05.010
